# Diurnal Variation of Tight Junction Integrity Associates Inversely with Matrix Metalloproteinase Expression in *Xenopus laevis* Corneal Epithelium: Implications for Circadian Regulation of Homeostatic Surface Cell Desquamation

**DOI:** 10.1371/journal.pone.0113810

**Published:** 2014-11-20

**Authors:** Allan F. Wiechmann, Brian P. Ceresa, Eric W. Howard

**Affiliations:** 1 Department of Cell Biology, University of Oklahoma Health Sciences Center, Oklahoma City, Oklahoma, United States of America; 2 Department of Ophthalmology, University of Oklahoma Health Sciences Center, Oklahoma City, Oklahoma, United States of America; 3 Department of Pharmacology and Toxicology, University of Louisville School of Medicine, Louisville, Kentucky, United States of America; Northwestern University, United States of America

## Abstract

**Background and Objectives:**

The corneal epithelium provides a protective barrier against pathogen entrance and abrasive forces, largely due to the intercellular junctional complexes between neighboring cells. After a prescribed duration at the corneal surface, tight junctions between squamous surface cells must be disrupted to enable them to desquamate as a component of the tissue homeostatic renewal. We hypothesize that matrix metalloproteinase (MMPs) are secreted by corneal epithelial cells and cleave intercellular junctional proteins extracellularly at the epithelial surface. The purpose of this study was to examine the expression of specific MMPs and tight junction proteins during both the light and dark phases of the circadian cycle, and to assess their temporal and spatial relationships in the *Xenopus laevis* corneal epithelium.

**Methodology/Principal Findings:**

Expression of MMP-2, tissue inhibitor of MMP-2 (TIMP-2), membrane type 1-MMP (MT1-MMP) and the tight junction proteins occludin and claudin-4 were examined by confocal double-label immunohistochemistry on corneas obtained from *Xenopus* frogs at different circadian times. Occludin and claudin-4 expression was generally uniformly intact on the surface corneal epithelial cell lateral membranes during the daytime, but was frequently disrupted in small clusters of cells at night. Concomitantly, MMP-2 expression was often elevated in a mosaic pattern at nighttime and associated with clusters of desquamating surface cells. The MMP-2 binding partners, TIMP-2 and MT1-MMP were also localized to surface corneal epithelial cells during both the light and dark phases, with TIMP-2 tending to be elevated during the daytime.

**Conclusions/Significance:**

MMP-2 protein expression is elevated in a mosaic pattern in surface corneal epithelial cells during the nighttime in *Xenopus laevis*, and may play a role in homeostatic surface cell desquamation by disrupting intercellular junctional proteins. The sequence of MMP secretion and activation, tight junction protein cleavage, and subsequent surface cell desquamation and renewal may be orchestrated by nocturnal circadian signals.

## Introduction

The uniquely transparent cornea comprises much of the anterior surface of the eye and is incessantly challenged by environmental assaults during the daytime. A non-keratinizing stratified squamous epithelium and thin layer of tear film cover the corneal surface. The single thin layer of cells at the tear/surface interface of the corneal epithelium (CE) provides the most crucial barrier to ocular infection. Deterioration of sight due to breakdown of corneal barrier integrity is a health concern for millions of people. Disruption of the integrity of the CE barrier contributes to incapacitating disorders such as herpetic infection and corneal erosions, and the very prevalent disorder of keratoconjunctivitis (dry eye syndrome) [1]–[4].

The basal layer of cells is the proliferative layer of the CE, and as the basal CE cells divide (on a cyclic rhythm) [5]–[8], they give rise to daughter cells that are displaced apically. These epithelial cells continue to be displaced apically as additional cells are generated from the basal epithelium. The addition of new cells from the basal layer is balanced by the shedding of living epithelial cells at the CE surface. In the healthy cornea, desquamation of surface CE cells is not dependent upon apoptosis as was once thought [9], indicating that the living surface cells must actively dissociate themselves (*i.e.*; disconnect their junctional complexes) from neighboring cells.

The transmembrane tight junction proteins occludin and claudin extend extracellularly from the lateral plasma membranes to form covalent bonds with complementary proteins on neighboring cells. This cross-linking creates a paracellular barrier that regulates the permeability of the epithelium, which is especially tight in the CE. Occludin and claudin have been shown to be somewhat unanticipated targets for cleavage by matrix metalloproteinases (MMPs) in a wide range of pathological conditions and tissues [10]–[19]. MMPs were named as such based on initial observations in *Xenopus laevis* in which they degraded extracellular matrix proteins such as collagen [20], [21], but it has become increasingly apparent that the array of protein targets of MMP cleavage extend far beyond extracellular matrix proteins [22]–[24]. The vast majority of studies on the role MMPs in adult tissues have focused on their responses to pathological situations [11]–[13], [19], [25]. In this study, we propose a novel role for MMP-2 (and its binding partners) in the normal homeostasis of epithelial renewal and turnover.

The mechanism of activation of MMP-2 is perhaps the best understood of the entire family of zinc-dependent MMPs [26]–[29]. The most potent means of activation of MMP-2 occurs through formation of a ternary complex with membrane type 1 (MT1)-MMP (also called MMP-14) and tissue inhibitor of MMP-2 (TIMP-2) [26]–[28]. TIMP-2 bound to the membrane-anchored MT1-MMP acts as a receptor for pro-MMP-2. Binding of pro-MMP-2 to TIMP-2 (bound to MT1-MMP) enables adjacent active molecules of MT1-MMP to cleave and activate the MMP-2. After MT1-MMP is activated, it is rapidly internalized from the cell surface [27], [28]. MMP-2 activity is highly dependent on the levels of TIMP-2; low (equimolar) levels of TIMP-2 are required for MMP-2 activation, whereas a higher (two-fold) level of TIMP-2 inhibits MMP-2 activation [28], [30].

We chose the *Xenopus laevis* model because our previous studies on circadian events in the *Xenopus* eye established the foundation for this present investigation, and circadian rhythms have been particularly well-studied in this model [31]–[33]. Also, since *Xenopus* are aquatic, there are fewer confounding issues of nocturnal eyelid closure and daytime dryness as occurs in terrestrial mammals. Additionally, the functions of MMPs have been particularly well-studied in this species in which they were originally discovered [20], [21], [34]. The purpose of this project was to determine if; 1) there are day/night changes in the pattern of expression of tight junction proteins in *Xenopus laevis* CE, 2) if any diurnal changes in the pattern of tight junction protein expression correlate negatively with local expression of MMP proteins, and 3) and if areas of surface cell desquamation are associated with the presence of MMP at or near the surface epithelium. Our data suggest that discrete clusters of surface CE are subjected to intercellular detachment and subsequent desquamation, and that this process is mediated via MMP activity associated with tight junction protein dissociation. Furthermore, this mosaic pattern of MMP expression, tight junction degradation and cell surface desquamation occurs preferentially during the nighttime, suggesting a circadian influence on CE surface cell homeostatic turnover.

## Materials and Methods

### Animals

Post-metamorphic *Xenopus laevis* (African clawed frogs) were obtained from Xenopus Express (Brooksville, FL) and maintained in aquaria at 20°C on a daily lighting schedule of 12 hr dark: 12 hr light for a minimum of two weeks. Frogs were deeply anesthetized by immersion in 0.5% triciane methanesulfonate (MS-222; Sigma, St. Louis, MO) in buffered water and killed by decapitation at specific times during the light/dark cycle.

### Ethics statement

Animals were cared for in accordance with the guidelines of the Public Health Service Policy on Humane Care and Use of Laboratory Animals and were approved by the Institutional Animal Care and Use Committee of the University of Oklahoma Health Sciences Center (Protocol number 12-076-T).

### Tissue preparation

Whole eye globes were removed from the frogs and immersion-fixed for a total of one hour at 4°C in 4% paraformaldehyde in 0.1 M phosphate buffer pH 7.4. Midway through the fixation period, the anterior segments (cornea, iris, ciliary body and lens) were dissected free of the posterior segments. This protocol was employed to minimize disturbance of the CE prior to fixation, yet achieve sufficient preservation of the cornea.

For preparation of cryostat sections, corneas were dissected free of the rest of the anterior segments and rinsed three times with 0.1 M phosphate-buffered saline (PBS), pH 7.4. Isolated corneas were transferred to 30% sucrose in phosphate buffer for 16–20 hr at 4°C, and then mounted in Tissue-Tek O.C.T. mounting matrix (Sakura Finetek, Torrance, CA). Sagittal 10 µm sections were cut on a cryostat microtome and collected onto glass slides. Sections were rinsed three times with PBS, and then placed into 0.01 M sodium citrate buffer with 0.05% Tween-20 (Sigma) for epitope retrieval treatment. Slides were incubated at 100°C for 10 min, allowed to cool to room temperature (RT) for 20 min, then rinsed three times with PBS and incubated in blocking buffer (2% bovine serum albumin [BSA; Sigma] 0.2% Triton X-100 [Sigma], and 0.04% sodium azide in PBS) for at least one hour.

For preparation of corneas for use in whole mount immunocytochemistry, fixed anterior segments were placed individually into shallow-bottomed 2.0 ml microfuge tubes. They were rinsed three times with PBS and then transferred into 0.01 M sodium citrate buffer/0.05% Tween-20 for epitope retrieval treatment. Specimens were incubated in a water bath at a starting temperature of 70°C which increased progressively to 80°C during a course of 10 min. The temperature range was selected to afford some epitope retrieval, yet minimize distortion of the tissues. Epitope retrieval was necessary in this study due to the relatively lower reactivity of *Xenopus* proteins with antibodies generated against mammalian proteins. The anterior segments were kept intact during this procedure as they helped to lessen distortion of the corneas during the heating process. Tissues were allowed to cool to RT for 30 min, then rinsed three times with PBS and incubated in BSA blocking buffer for at least two hours. The corneas were dissected free of the remaining anterior segment components during the PBS washing steps.

### Confocal immunocytochemistry procedures

Cryostat sections or whole corneas were incubated with 5 µg/ml of primary antibody in BSA blocking buffer for three days at 10°C. This prolonged incubation period was necessary because of the relatively lower reactivity of the mammalian antibodies with the *Xenopus* proteins. The primary antibodies used in this study are listed in Table 1. For negative controls, tissue sections or whole corneas were incubated in blocking buffer lacking the primary antibody. Following incubation with the primary antibody, sections or whole corneas were rinsed three times in PBS, and incubated in 2 µg/ml of goat anti-rabbit or goat anti-mouse antibody conjugated to AlexaFluor 488 (green; Molecular Probes, Eugene, OR) or AlexaFluor 568 (red) in PBS for 2 hr at RT. Sections or whole corneas were rinsed three times in PBS.

**Table 1:** List of Primary Antibodies.

Antigen	Manufacturer (catalog #)	Host
Claudin-4	Invitrogen (364800)	rabbit
Claudin-4	Invitrogen (329400)	mouse
MMP-2	Invitrogen (436000)	mouse
MT1-MMP, hinge region	Millipore (AB6004)	rabbit
Occludin	Invitrogen (711500)	rabbit
Occludin	Invitrogen (331500)	mouse
TIMP-2	Millipore (AB2965)	rabbit
TIMP-2	Millipore (MAB13446)	mouse

Whole corneas were mounted (CE surface up) onto glass slides by making 4–5 slits from peripheral to mid-cornea with scissors and then gently compressing the tissue under weighted coverslips after the mounting matrix was applied to achieve a flat-mounted cornea. Coverslips were mounted onto the slides with tissue sections or whole corneas with Prolong Gold Antifade reagent containing DAPI (Life Technologies, Eugene, OR).

For double-label immunocytochemistry, the same procedure was followed as described for the primary antibody incubations, except that after the first labeling procedure, the tissues or sections were incubated with 5 µg/ml of a second primary antibody that was raised in a species different from that used in the first immunolabeling. The primary antibody was labeled with 2 µg/ml goat anti-rabbit or goat anti-mouse antibody conjugated to AlexaFluor 488 (green) or AlexaFluor 568 (red), using the same conditions as described for the first immunolabeling, and then mounted onto glass slides and coverslips. In all of the corneal whole mount experiments, images from only the central region of the cornea were captured so that similar regions among samples were analyzed.

The immunolabeled sections and corneal whole mounts were viewed by confocal microscopy, using an Olympus FluoView 1000 laser-scanning confocal microscope (Center Valley, PA). The pinhole (confocal aperture diameter) conditions were fixed at 105 µm in all of the images generated in this study. The objective lenses used in this study were an Olympus PlanApo N 60×/1.42 Oil lens (∞/0.17/FN26.5), and UPlanSApo 100×/1.40 Oil lens (∞/0.17/FN26.5). The control specimens were examined under identical conditions to the appropriate non-control specimens. In all cases, image scale was calibrated and brightness and contrast were adjusted as necessary to highlight specific labeling.

### Data analysis

Red or green channels of confocal images were converted to grayscale in Photoshop for quantitative analysis. Relative image intensities of four representative areas of each group were measured using Image J software. Statistical analysis was performed using the unpaired Student's t-test (GraphPad Prism 5.0 software, San Diego, CA). Significance was assumed for *p* values less than 0.05.

### *In situ* zymography procedures

MMP activity in *Xenopus* CE was analyzed by *in situ* zymography, as described by Hadler-Olsen et al. [35] DQ gelatin (Molecular Probes) is a fluorescein-gelatin conjugate that is so heavily labeled with fluorescein that the fluorescence is quenched. This substrate is digested by gelatinases and collagenases in tissue sections to yield fluorescent peptides that can be visualized by fluorescence microscopy. Corneas were obtained from frogs late in the dark period 2 hrs *before* lights on, and early in the light period 2 hrs *after* lights on. Isolated corneas were mounted in O.C.T. mounting matrix, and sagittal 10 µm sections were cut on a cryostat microtome and collected onto glass slides. Unfixed corneal cryostat sections were air-dried and stored at -80°C until time of use.

Thawed sections were rinsed once with reaction buffer (50 mM Tris-HCl, 150 mM NaCl, 5 mM CaCl_2_, and 0.2 mM sodium azide, pH 7.7), and then incubated in a humidified chamber at RT for 30 min in reaction buffer containing 1 mg/ml DQ-gelatin with or without 20 mM EDTA or 1 mM 1,10-phenanthroline (Sigma) as negative controls. EDTA and phenanthroline inhibit MMP activity, and are often employed to determine the specificity of the gelatinase activity [35], [36]. The sections were then rinsed three times in PBS, and placed into 4% paraformaldehyde in 0.1 M phosphate buffer pH 7.4 for 10 min at RT. The sections were rinsed three times in PBS, and coverslips were mounted onto the slides with Prolong Gold Antifade reagent containing DAPI. The presence of fluorescence in the corneal sections was analyzed by confocal microscopy.

## Results

### MMP-2 and TIMP are co-localized in *Xenopus* surface corneal epithelium sections

**Figure 1:**
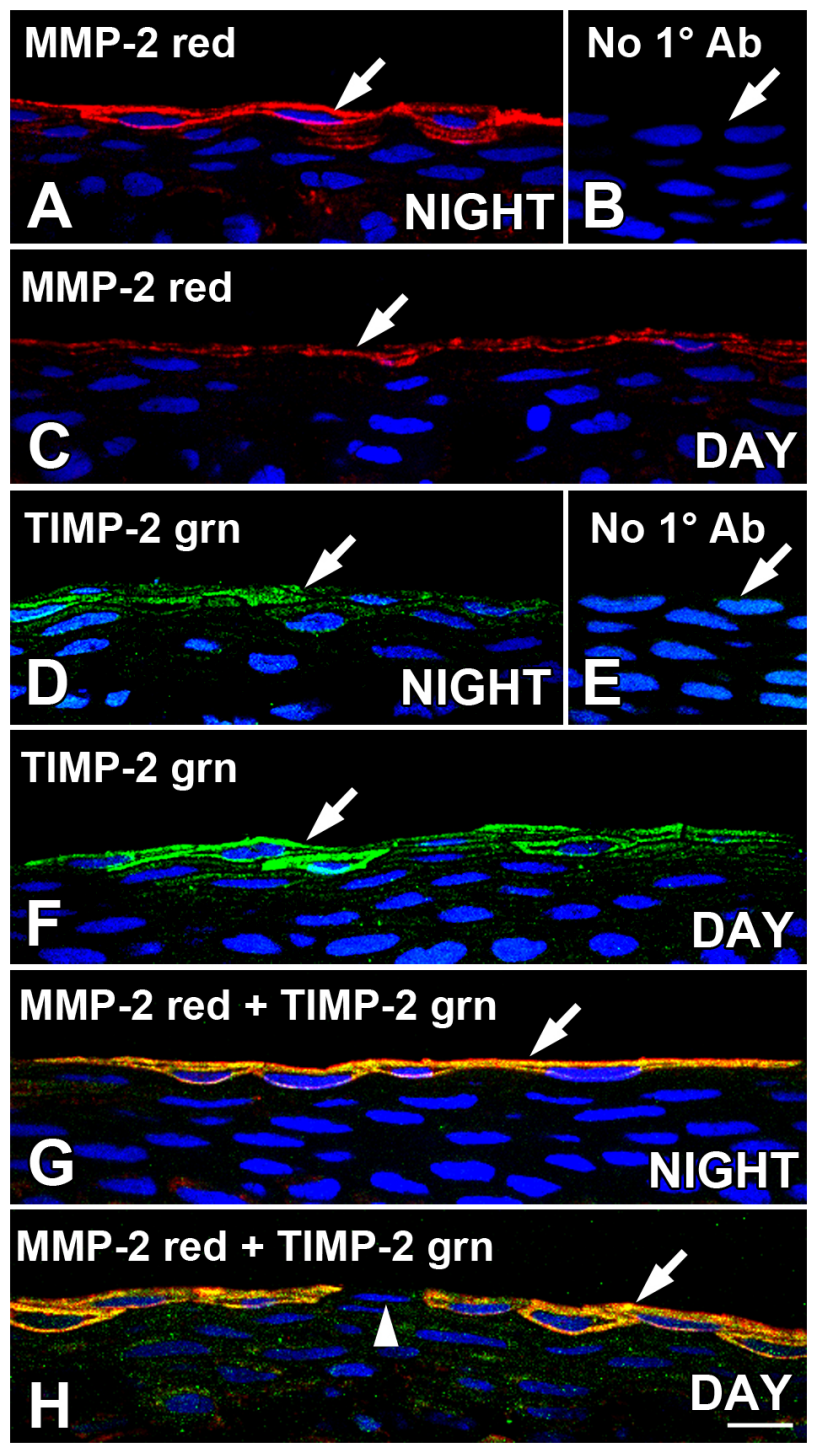
MMP-2 and TIMP are co-localized in *Xenopus leavis* surface corneal epithelium sections. Cryostat sections of corneas obtained during the late dark period (NIGHT: two hrs *before* lights on) or early light period (DAY; two hrs *after* lights on) were immunolabeled with MMP-2 and or/TIMP-2 antibodies and analyzed with confocal microscopy. (**A**) At nighttime, MMP-2 immunoreactivity (red) was localized in cell clusters mainly to the surface layer of CE cells (arrow), with some labeling also present in the underlying sub-superficial layer of cells. Immunolabeling in the deeper layers of the CE was very sparse. (**B**) As negative controls, corneal sections obtained at night were processed for immunohistochemistry in the absence of primary antibody, and no specific immunoreactivity was observed (arrow). (**C**) At early daytime, intensity of MMP-2 immunoreactivity (red) was generally diminished relative to the night time levels (arrow). (**D**) At late nighttime, TIMP-2 immunoreactivity (green) was localized in cell clusters mainly to the surface layer of CE cells (arrow), with some labeling also present in the underlying sub-superficial layer of cells. Immunolabeling in the deeper layers of the CE was very sparse. (**E**) As negative controls, corneal sections obtained during the daytime were processed for immunohistochemistry in the absence of primary antibody, and no specific immunoreactivity was observed (arrow). (**F**) At early daytime, intensity of TIMP-2 immunoreactivity (green) was generally higher relative to the nighttime levels (arrow), and some labeling also present in the underlying sub-superficial layer of cells, inverse of the temporal pattern observed with MMP-2 in A and C. (**G**) Double-label immunocytochemistry of MMP-2 (red) and TIMP-2 (green) of nighttime corneas revealed co-localization of the two proteins. Yellow indicates regions of co-localization of the red and green signal (arrows). (**H**) During the early daytime, MMP-2 (red) and TIMP-2 (green) displayed a similar yellow co-localization (arrow) as seen at nighttime. Note that the two proteins were either expressed together (arrow) or not at all (arrowhead). Sections were stained with DAPI, which stained the nuclei blue. Scale bar = 20 µm.

Cryostat sections of *Xenopus* cornea immunolabeled for either MMP-2 or TIMP-2 revealed that both proteins were expressed mainly in the squamous surface layer of cells in the CE (Figure 1). The experiments with corneal sections preceded the use of corneal whole mounts in this study, and during the course of the study, we altered the timing of harvesting the corneas. The corneas used for the cryostat section experiments were obtained either late in the dark period (2 hr *before* lights on) or early in the light period (2 hrs *after* lights on), so the time points were only four hours apart. These were the same time points used in the *in situ* zymography experiments, but later when using corneal whole mounts, we obtained them at time points that were 12 hours apart in the diurnal cycle, as will be described in that section.

The MMP-2 and TIMP-2 immunolabeling appeared to be associated both with the plasma membrane and cytoplasm of surface CE cells (Figure 1). It is important to note that the immunolabeling of these proteins was not continuous throughout the cornea; they were present sporadically in clusters of surface cells. Some intermittent immunolabeling for both proteins was also observed in the layer of sub-superficial cells directly below the surface layer. When present in the scattered cell clusters, the intensity of immunolabeling for MMP-2 was generally higher late at night (Figure 1A) than early in the day (Figure 1C), whereas TIMP-2 labeling intensity was generally lower late at night (Figure 1D) than early in the day (Figure 1F). Sections incubated without primary antibody displayed no specific immunoreactivity (Figure 1B and 1E). Double-label immunocytochemistry of MMP-2 and TIMP-2 revealed that the two proteins co-localized to the same cells in the surface CE (Figure 1G and 1H). Moreover, they were either expressed together or neither protein was expressed.

### MMP gelatinase activity is present in *Xenopus* surface corneal epithelium

**Figure 2:**
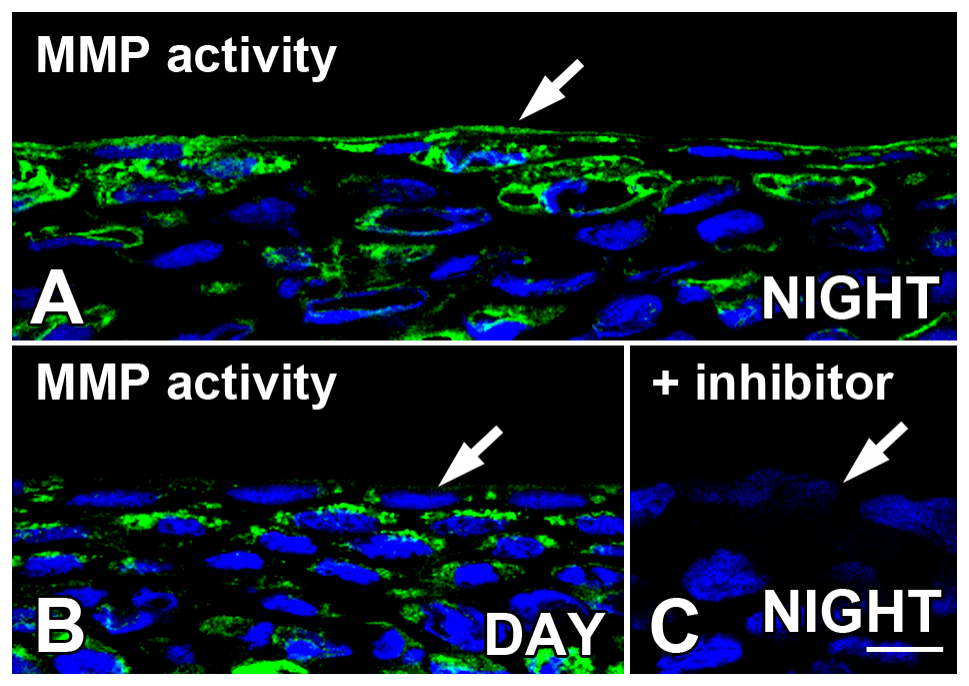
*In situ* zymography demonstrates MMP gelatinase activity in *Xenopus* surface corneal epithelium. The presence of green fluorescence with confocal microscopy indicates the presence of gelatinase enzyme activity in unfixed cornea sections obtained during the late dark period (NIGHT: two hrs *before* lights on) or early light period (DAY; two hrs *after* lights on). (**A**) Late at night, MMP activity (green) was present in the surface layers of the CE (arrow) with some labeling also present in the deeper layers of the CE. In the early daytime, MMP activity (green) in the surface CE (arrow) appeared to be lower early in the light period with some labeling persisting in the deeper layers of the cornea. (C) Inclusion of a specific inhibitor of MMP-2 and MMP-9 (1.0 µm phenanthroline) in the incubation mixture blocked most of the gelatinase activity. Sections were stained with DAPI, which stained the nuclei blue. Scale bar = 20 µm.

*In situ* zymography was performed on unfixed *Xenopus* corneal sections, in which a quenched fluorescently-labeled gelatin complex is cleaved by endogenous active gelatinases (*i.e.*; MMP-2 and MMP-9) to un-quench the fluorescent signal. Therefore, the presence of green fluorescence indicates the presence of gelatinase enzyme activity. High levels of presumptive MMP activity were present in the surface layers of the CE, especially late at night (Figure 2A). The endogenous MMP activity appeared to be lower early in the light period (Figure 2B), thus supporting the hypothesis that MMP activity declines during the light period. Inclusion of a specific inhibitor of MMP-2 and MMP-9 (phenanthroline) in the incubation mixture blocked most of the gelatinase activity (Figure 2C), confirming that most of the fluorescent signal is due to the presence of MMP activity. In addition, the presence of the zinc chelator EDTA blocked the gelatinase activity (data not shown), also suggesting that the observed gelatinase activity is due to MMP.

### Differential expression of tight junction proteins and MMPs in whole-mounted corneas

Immunocytochemistry was performed on flatmount preparations of *Xenopus* corneas that were obtained from animals in the late afternoon (9 hours *after* lights on during a 12L:12D cycle; CT09), and in the late night (3 hours *before* lights on; CT21). These times points (12 hrs apart) were selected based on our prediction that maximal tight junction disruption would occur late in the dark period (having a more prolonged period exposed to nighttime signals), and that tight junctions would be least disrupted late in the day, since they were further temporally separated from the nighttime signaling. Other intermediate time points were also analyzed and determined to exhibit a range of intermediate characteristics of the late evening and late afternoon time points (data not shown). The most obvious day/night differences were between the late night and late afternoon time points, which are reported here.

### Occludin and claudin-4 are co-localized on surface corneal epithelium lateral membranes and are disrupted at night

**Figure 3:**
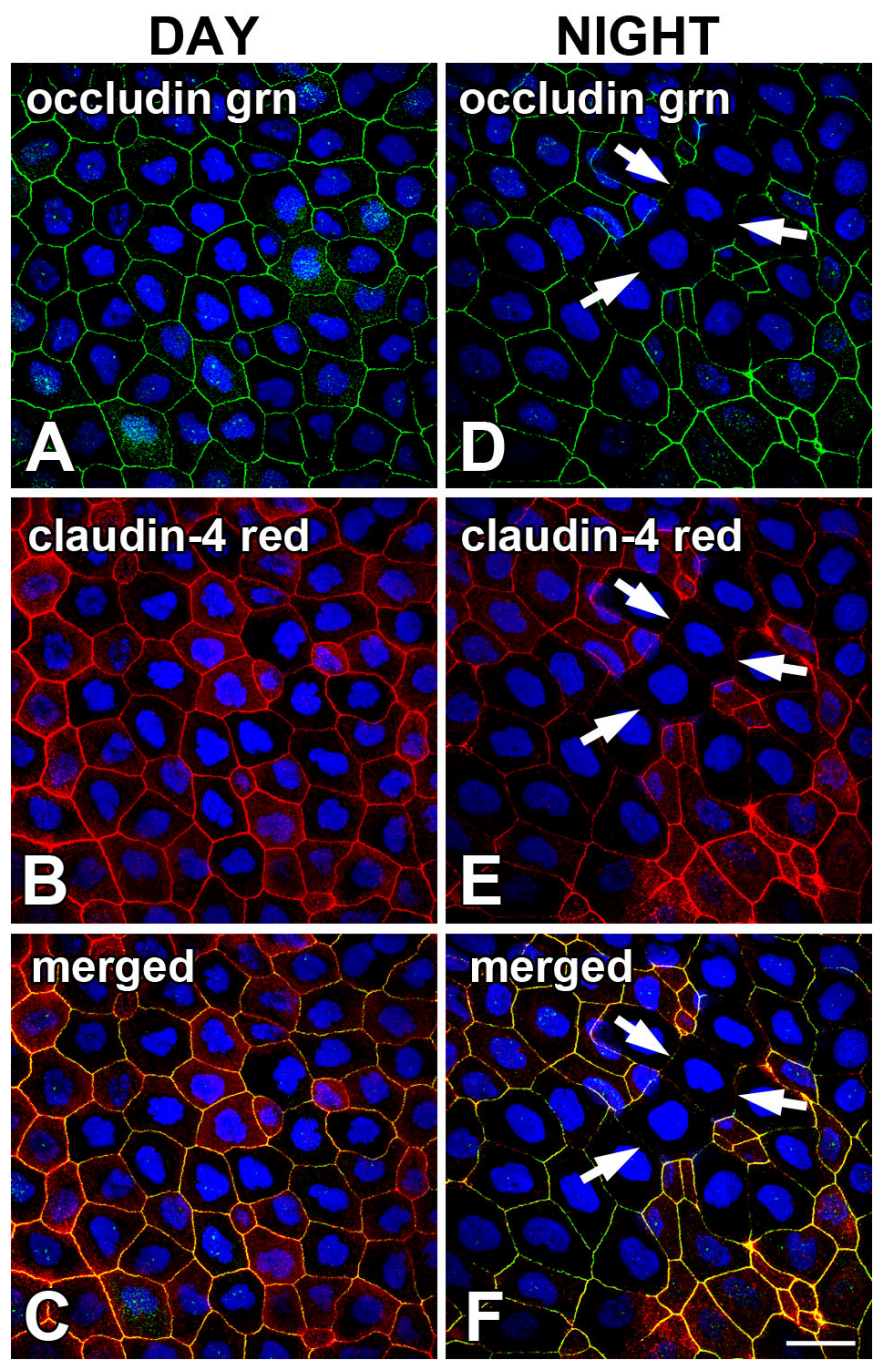
Tight junction proteins occludin and claudin-4 are co-expressed in *Xenopus* corneal epithelium lateral membranes and are disrupted at night. Double-label confocal immunocytochemistry was performed on whole flat-mounted preparations of *Xenopus* corneas that were obtained from animals in the late afternoon (DAY; 9 hours *after* lights on in a 12L:12D cycle) and in the late night (NIGHT; 3 hours *before* lights on). (**A**) In the late afternoon, occludin (green) was uniformly primarily localized to the lateral membranes of the surface CE. (**B**) The same specimen as in A was labeled for the presence of claudin-4 (red), and was also uniformly primarily localized to the lateral membranes of the surface CE. (**C**) Merged green/red images from A and B demonstrate a high degree of co-localization in the surface cell CE lateral membranes, as indicated by the yellow signal. (**D**) In the late night, occludin (green) was localized to the CE surface cell lateral membranes as during the day, but the pattern of labeling was often interrupted in some clusters of cells (arrows). (**E**) The same specimen as in D was labeled for the presence of claudin-4 (red), and was also localized to the CE surface cell lateral membranes, but the pattern of labeling was also interrupted in the same clusters of cells (arrows). (**F**) Merged green/red images from D and F demonstrate a high degree of co-localization in the surface cell CE lateral membranes, as indicated by the yellow signal, with a similar level of disruption a discrete loci. Specimens were stained with the blue nuclear DAPI stain. Scale bar = 20 µm.

At the late afternoon time point (3 hrs before lights off) occludin and claudin-4 were co-localized to the lateral membranes of the surface CE as predicted (Figure 3A–C). The immunolabeling pattern showed continuous bands encircling the apical region of the lateral membranes of the surface layer of CE cells. Although the degree of co-localization was very high, subtle differences in relative intensities of occludin and claudin-4 labeling were often apparent (Figure 3C). This suggests that the relative expression of the two proteins is not precisely the same in all cells, although they are generally quite consistent.

In contrast, at the late evening time point (3 hrs before lights on) occludin and claudin-4 immunolabeling often displayed a disruption of the daytime pattern in small discrete clusters of cells (Figure 3D–F). The loss of occludin and claudin-4 on some lateral membranes suggested that the tight junction barrier was disrupted at these specific locations. There appeared to be more variation in the relative degree of co-localization and differential expression of occludin and claudin-4 on the intact lateral membranes compared to what was observed during the day (Figure 3C, F).

### Occludin and claudin-4 expression appear inversely associated with the presence of MMP-2 on the corneal epithelium surface and exhibit day/night changes

**Figure 4:**
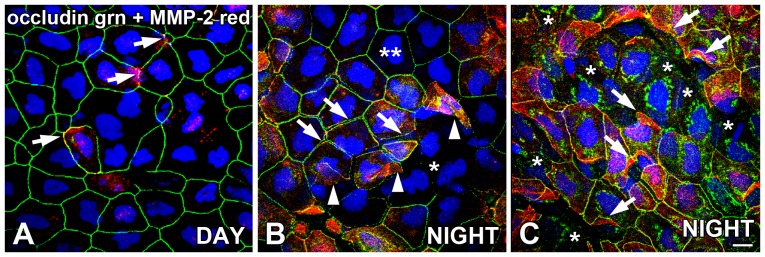
Occludin expression on *Xenopus* surface CE is disrupted at night, and is inversely associated with MMP-2 expression. Flat-mounted whole corneas were double-labeled for localization of occludin and MMP-2 immunoreactivity in the late afternoon (DAY; 9 hours *after* lights on in a 12L:12D cycle) and in the late night (NIGHT; 3 hours *before* lights on). (**A**) In the late afternoon, occludin (green) immunolabeling of the lateral membranes of the surface CE was generally intact, and some sporadic labeling of MMP-2 (red) was also present on the lateral membranes and/or cytoplasm (arrows) which was sometimes co-localized (yellow) with occludin. (**B**) During the late night, most of the surface CE cells exhibited intact occludin (green) labeling on their lateral membranes (double asterisks), but there were also many clusters of surface CE cells that lacked occludin immunoreactivity in their lateral membranes (single asterisk). In neighboring cells of some occludin-negative clusters, there was a gap between the occludin-labeled lateral membranes, indicating the disruption of tight junctions between the cells (arrows). MMP-2 (red) was very often expressed in the cells that exhibited gaps between the lateral membranes. Also, in these areas of high MMP-2 expression, upward-folding flaps of surface cells were lifting from the CE surface, representing cells in the act of desquamation (arrowheads). (**C**) In some cell clusters that were almost devoid of occludin labeling on their lateral membranes at nighttime (presumptive former sub-superficial cells; asterisks), intense occludin (green) immunoreactivity was observed in perinuclear intracytoplasmic compartments. The neighboring surface CE cells express high levels of MMP-2 (red) that are in the process of desquamation (arrows). There was also considerable co-localization (yellow) of occludin and MMP-2 on the lateral membranes between neighboring cells. Specimens were stained with the blue nuclear DAPI stain. Scale bar = 20 µm.

Flat-mounted whole corneas were double-labeled for the presence of occludin and MMP-2 immunoreactivity at late daytime (3 hrs before lights off) and nighttime points (3 hrs before lights on). At the late afternoon time point, occludin immunolabeling of the lateral membranes was generally intact (Figure 4A) as described earlier (Figure 3A). In some daytime specimens, we occasionally observed some sparse scattered MMP-2 immunoreactivity located in both the cytoplasm and/or lateral membranes of the surface CE, which was sometimes co-localized with occludin (Figure 4A). These discrete sites of MMP-2 immunoreactivity often appeared to coincide with some loss of occludin immunolabeling of the lateral membranes.

At the late night time point, most of the surface CE cells exhibited intact occludin labeling, although there were many clusters of surface CE cells that lacked occludin immunoreactivity in their lateral membranes (Figure 4B), as described earlier (Figure 3D). They were considered to be the former sub-superficial layer of CE cells that had been only recently exposed to the corneal surface and had not yet expressed occludin in their lateral membranes. In clusters bordering these occludin-negative cell clusters, the pattern of occludin labeling was often disrupted, insofar as the cells had occludin on their lateral membranes, but the cells were clearly not attached to their neighboring cells (Figure 4B). Concomitant with this disruption of intercellular occludin attachments between the lateral membranes of neighboring cells, MMP-2 immunoreactivity was quite elevated in both the cytoplasm and lateral membranes of these cells (Figure 4B, C). Many of these MMP-2 immunoreactive cells displaced upward-folding flaps of membrane, indicative of cells that were in the process of desquamation (Figure 4B, C).

In the cell clusters that were almost devoid of occludin labeling on their lateral membranes, intense occludin immunoreactivity was often observed in perinuclear intracytoplasmic compartments (Figure 4C). We speculate that this intracytoplasmic occludin may represent newly-synthesized occludin that has not yet been transported to the plasma membrane, and that these cells may represent a population of sub-superficial cells recently exposed to the surface because of the recent desquamation of the overlying surface CE cells, and are undergoing a rapid terminal maturation process. Often, the bordering clusters of surface CE cells exhibited very high levels of MMP-2 immunoreactivity in their cytoplasm and lateral membranes, and were obviously in the process of desquamation (Figure 4C). Therefore, at nighttime in discrete clusters of surface CE cells, there was disruption of occludin labeling of the lateral membranes, a concomitant high incidence of MMP-2 immunoreactivity, and subsequent surface cell desquamation.

**Figure 5:**
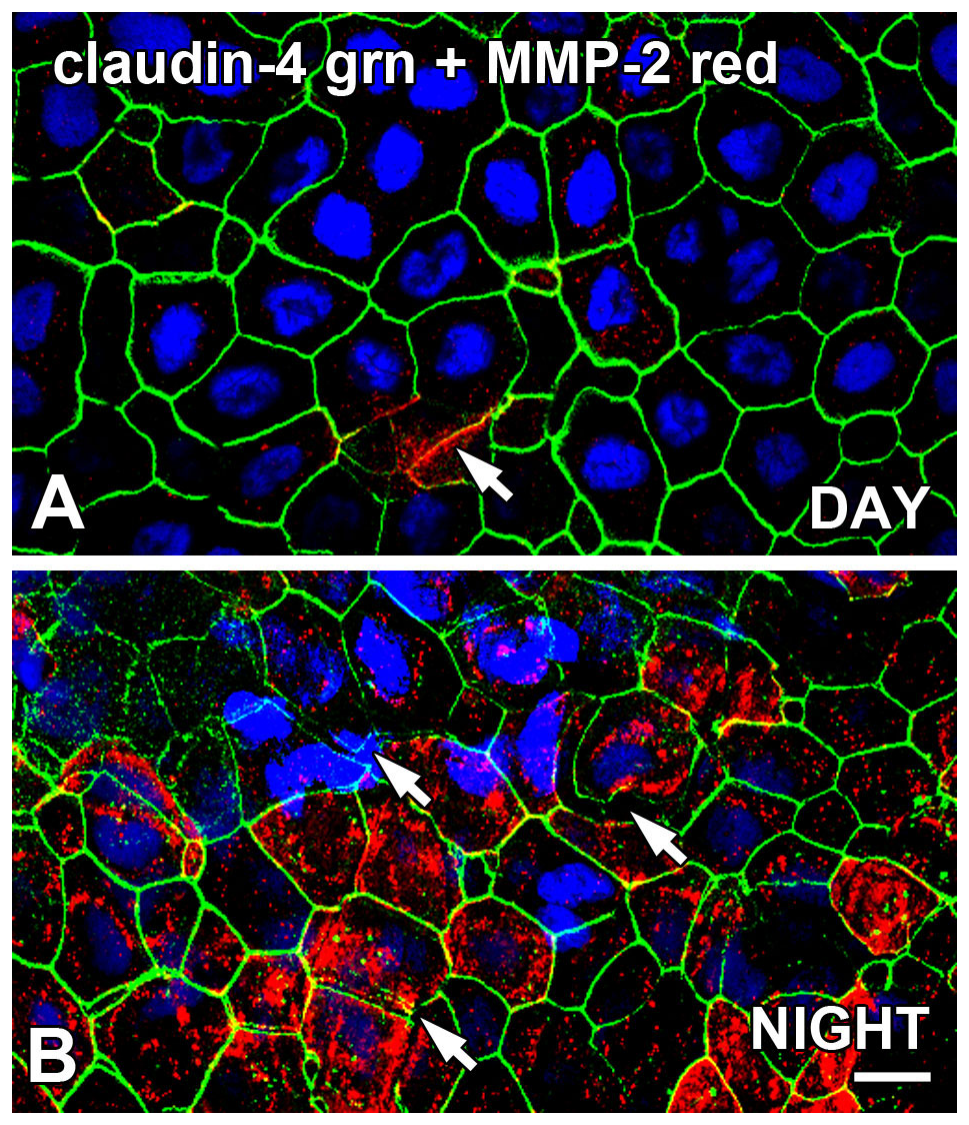
Claudin-4 expression on *Xenopus* surface CE is disrupted at night, and is inversely associated with MMP-2 expression. Flat-mounted whole corneas were double-labeled for localization of claudin-4 and MMP-2 immunoreactivity in the late afternoon (DAY; 9 hours *after* lights on in a 12L:12D cycle) and in the late night (NIGHT; 3 hours *before* lights on). (**A**) In the late afternoon, claudin-4 (green) immunolabeling of the lateral membranes of the surface CE was generally intact, and some sporadic labeling of MMP-2 (red) was also present on the lateral membranes and/or cytoplasm (arrows) which was sometimes co-localized (yellow) with occludin. (**B**) During the late night, most of the surface CE cells exhibited intact claudin-4 (green) labeling on their lateral membranes, but there were also many clusters of surface CE cells in which the claudin-4 immunoreactivity was disrupted (arrows) and was accompanied by high levels of MMP-2 immunoreactivity. Specimens were stained with the blue nuclear DAPI stain. Scale bar = 20 µm.

Similar to the inverse pattern of occludin and MMP-2 association at the surface CE at nighttime, claudin-4 and MMP-2 expression appeared to be inversely associated and exhibited day/night changes (Figure 5). During the daytime, claudin-4 immunoreactivity (Figure 5A) was very comparable to the intact occludin labeling of the surface CE lateral membranes observed during the day (Figure 4A) which was relatively unperturbed. Some occasional scattered MMP-2 immunoreactivity was exhibited in both the cytoplasm and lateral membranes during the day, and the membrane localization was either co-localized with claudin-4 or occurred in the absence of claudin-4 immunoreactivity (Figure 5A). This pattern is identical to the inverse association seen with occludin and MMP-2 (Figure 4A). The sites of membrane-localized MMP-2 were often associated with some disruption of claudin-4 membrane localization (Figure 5A). At nighttime, claudin-4 immunolabeling present in clusters of the CE surface cell lateral membranes was disrupted in the same pattern as occludin (Figure 4B, C); membrane labeling was sometimes discontinuous or absent (Figure 5B). Claudin-4 membrane labeling was also not contiguous, insofar as there was clear separation of neighboring cells even when claudin-4 was present on the membrane of both cells (Figure 5B). The disruption of intercellular attachment and claudin-4 on the lateral membranes was also associated with high expression levels of MMP-2 at nighttime.

### Preservation of occludin expression appears positively associated with TIMP-2 on surface corneal epithelium and exhibits day/night changes

**Figure 6:**
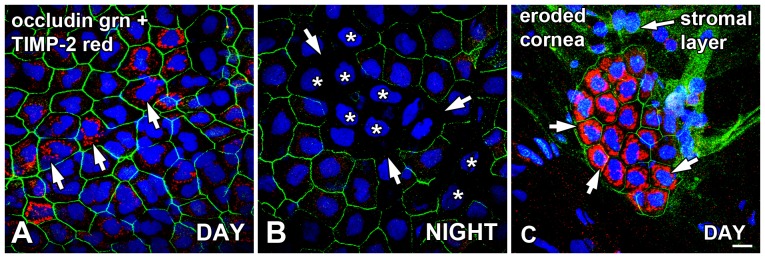
Preservation of occludin expression is positively associated with TIMP-2 on surface CE and exhibits day/night changes. Flat-mounted whole corneas were double-labeled for localization of claudin-4 and TIMP-2 immunoreactivity in the late afternoon (DAY; 9 hours *after* lights on in a 12L:12D cycle) and in the late night (NIGHT; 3 hours *before* lights on). (**A**) During the daytime, occludin (green) immunolabeling of the CE surface cell lateral membranes was generally intact. Small clusters of surface and sub-superficial CE cells exhibited TIMP-2 immunoreactivity in intracytoplasmic compartments (arrows). (**B**) At nighttime, TIMP-2 (red) immunoreactivity in the surface CE was very low, and occludin (green) immunoreactivity was disrupted (arrows) in some cell clusters (asterisks). (**C**) A cornea retrieved in the late afternoon (DAY) had an unidentified pathology in which almost all CE was eroded, leaving the stromal layer at the corneal surface. A small patch of about 20 CE cells persisted as a single monolayer above the stromal layer. The surviving CE cells displayed occludin (green) immunolabeling on their lateral membranes, and very intense immunolabeling of TIMP-2 (red) in their cytoplasm (arrows). There was some high non-specific green labeling of the connective tissue surface. Specimens were stained with the blue nuclear DAPI stain. Scale bar = 20 µm.

In contrast to the inverse association of occludin immunolabeling on CE surface cell lateral membranes with MMP-2 expression, TIMP-2 appeared to correlate positively with the preservation of occludin junction integrity. Although the ternary complex binding of TIMP-2 with MMP-2 and MT1-MMP is needed for maximal activation of MMP-2, a higher level of TIMP-2 inhibits MMP-2 activity [27], [28], [30]. High expression levels of TIMP-2 may therefore represent areas in which potential MMP-2-induced tight junction protein cleavage is attenuated. During the daytime, small clusters of surface and sub-superficial CE cells exhibited TIMP-2 immunoreactivity in intracytoplasmic compartments both in the surface layer of CE cells and in the sub-superficial layer of cells directly underneath the surface layer (Figure 6A). These clusters of cells were typically associated with intact occludin lateral membrane immunolabeling, as was seen in most of the CE during the daytime. At nighttime however, TIMP-2 immunoreactivity in the surface CE was very low, most notably in areas in which occludin immunolabeling of the surface CE lateral membranes was disrupted (Figure 6B).

During the course of this study, we occasionally encountered some frog corneas in which the CE was eroded due to unknown pathologies or injuries. These corneas were typically discarded, since the CE was generally absent. However, in one specimen that was immunolabeled for occludin and TIMP-2, we observed a small patch of CE cells resting above the stromal layer (Figure 6C). The CE cells in this patch of about 20 cells existed as a monolayer; the epithelium was no longer stratified. These surviving CE cells displayed robust occludin labeling on their lateral membranes, and extraordinarily intense immunolabeling of TIMP-2 in their perinuclear cytoplasm (Figure 6C). This fortuitous observation suggests that elevated TIMP-2 expression may aid in the preservation of tight junction integrity in pathologies or trauma to the CE.

### Membrane type 1-MMP is expressed in cytoplasmic compartments of surface corneal epithelium cells and associates with lateral membranes at nighttime

**Figure 7:**
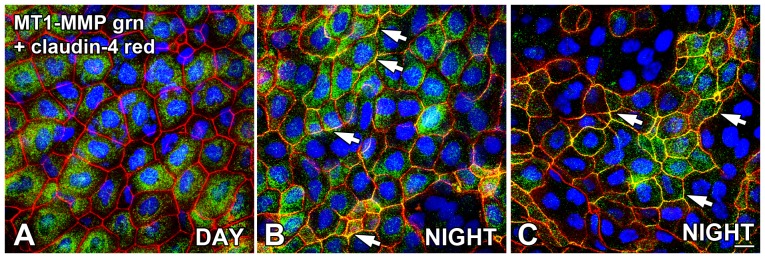
Membrane type 1-MMP is expressed in surface CE cells and associates with lateral membranes at nighttime. Flat-mounted whole corneas were double-labeled for localization of claudin-4 and membrane type 1-MMP (MT1-MMP) immunoreactivity in the late afternoon (DAY; 9 hours *after* lights on in a 12L:12D cycle) and in the late night (NIGHT; 3 hours *before* lights on). (**A**) In the late afternoon, claudin-4 (red) immunolabeling of the surface CE cell lateral membranes was generally intact and uniform. MT1-MMP (green) labeling, when present, was observed almost exclusively in cytoplasmic compartments of the surface CE. (**B, C**) Two examples are presented to illustrate that during the late night, a majority of the surface CE cells exhibited intact claudin-4 (green) labeling on their lateral membranes, but there were also many clusters of surface CE cells in which the claudin-4 immunoreactivity was disrupted. Also at nighttime, MT1-MMP labeling was present both in the cytoplasm and intermittently associated with the surface CE cell lateral membranes (arrows). The membrane co-localization of the red claudin-4 and green MT1-MMP resulted in a merged yellow labeling of the lateral membranes in some areas (arrows). Specimens were stained with the blue nuclear DAPI stain. Scale bar = 20 µm.

Membrane type-1 MMP (MT1-MMP; MMP-14) is the membrane-bound binding partner that forms a ternary complex with TIMP-2 and MMP-2 to cleave the pro-MMP-2 into the enzymatically-active MMP-2 [27], [28]. MT1-MMP was present in most, but not all surface CE cells, and was localized to cytoplasmic compartments both during the day and at night (Figure 7). At nighttime, however, MT1-MMP was often also localized to the surface CE cell lateral membranes (Figure 7B, C). The presence of MT1-MMP lateral membrane labeling was often associated with areas of tight junction disruption and surface cell desquamation as assessed by the diminished claudin-4 immunoreactivity.

### MMP-2 and TIMP-2 expression is significantly higher and lower at nighttime, respectively, in the surface corneal epithelium

**Figure 8:**
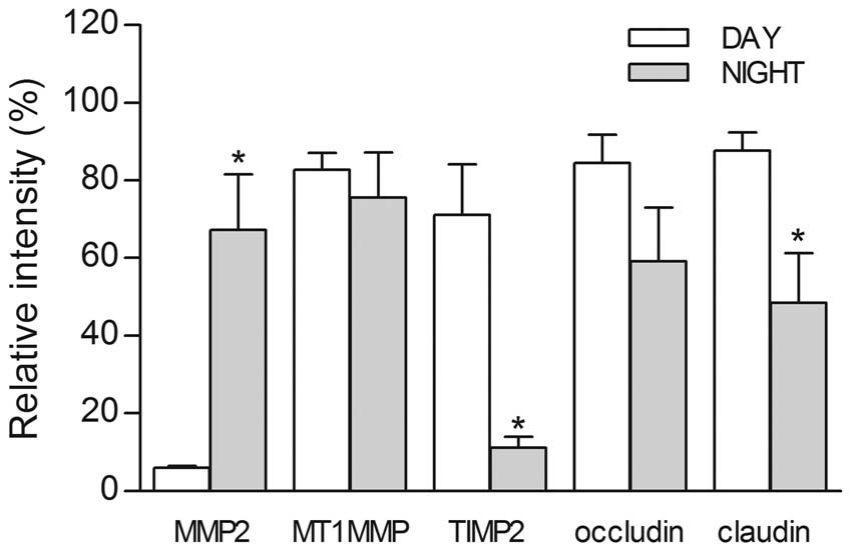
MMP-2 and TIMP-2 expression levels are inversely correlated during the light/dark cycle in surface corneal epithelium. Representative confocal images (N = 4) of each sample group were analyzed for day/night changes in immunolabeling intensity. MMP-2 immunolabeling intensity was significantly higher at nighttime than during the day (P<0.01), whereas TIMP-2 intensity was significantly lower at nighttime compared to the day (P<0.01; Two-tailed Student's t-test). MT1-MMP levels appeared unchanged between the light and dark cycle. The lower nighttime levels of occludin was not statistically significant, although the day/night change in claudin-4 expression was significant (P<0.05).

Quantitative analysis of day/night differences in MMP-2, TIMP-2, MT1-MMP, occludin, and claudin-4 expression revealed statistically significant differences for MMP-2 and TIMP-2, but not for MT1-MMP (Figure 8). MMP-2 expression during the night was about 10-fold higher than during the day (P<0.01) whereas TIMP-2 expression during the night was about 85% lower compared to daytime levels (P<0.01). MT1-MMP expression levels were approximately equivalent during the day and night, although as noted above in figure 7, much of the labeling at night was present on the lateral membranes. The expression levels of occludin and claudin-4 proteins were about 50–70% lower at nighttime than during the day, but the difference in occludin expression was not statistically significant although day/night change in claudin-4 expression was significant (P<0.05).

## Discussion

The corneal epithelium (CE) is a stratified epithelium that provides a major protective barrier to pathogens and environmental assaults on the eye. Disruption of the CE barrier function by disease or trauma and subsequent infections may be due in part to degradation of interconnecting tight junctions at the corneal surface by an induced secretion of matrix metalloproteinases (MMPs) as part of an inflammatory response [17], [37]–[41]. In contrast, we propose that regulated disruption of intercellular attachments of the living surface CE cells is required for normal homeostatic cell turnover. We hypothesize that daily desquamation of surface CE cells requires cleavage of transmembrane junctional proteins by MMPs secreted by CE cells in a circadian manner, whereas over-expression of MMPs in pathological conditions can be detrimental to the protective barrier function of the CE.

### Methodological approach of mosaic corneal epithelium cell turnover

During the course of this study, it became apparent that the surface CE cells are not all behaving synchronously. It is generally appreciated that cells of the surface layer of the CE do not detach all at the same time; this would logically be detrimental to the health of the cornea, and it is clear that the surface epithelium does not peel off together as contiguous sheets of cells [42]. There is experimental evidence in mammalian models that demonstrates that living, non-apoptotic surface CE cells are shed into the tear fluid either singly or as small clusters [6], [9]. Based on our observations of the *Xenopus* cornea, we propose that the cells of the surface CE are arranged into small mosaic clusters that differentiate, function, and desquamate in unison, but in a temporal sequence that is often out of phase with neighboring cell groups.

The concept that surface cell turnover occurs temporally as discrete mosaic clusters profoundly affects the methodological approaches we can use to effectively document the progression of periodic surface cell desquamation. The surface layer of cells of the CE represents a very small sub-population (probably less than 5%) of the entire population of CE cells. This is based on counting the number of cell nuclei in the entire thickness of CE, compared to the number cell nuclei at the surface (data not shown). Furthermore, we estimate that less than 10% of the surface layer of cells (*i.e.*; less than 0.5% of the total CE cells) are undergoing the final sequence of events that leads to imminent desquamation during the peak time period. Therefore, our *in vivo* analysis of surface CE had to be conducted primarily by observing the range of behaviors of the various clusters or populations of cells at the CE surface. Moreover, the inherently subjective nature of these analyses precluded the utility of more objective independent quantitative measurements, such as Western blotting or gel zymography. The necessary descriptive nature of the data presented in this report is a reflection of these methodological constraints. However, quantitative analyses of confocal images were performed to ascertain some statistically significant day/night differences in MMP-2 and TIMP-2 expression.

### Matrix metalloproteinase expression in corneal disease

In some epithelia, surface cells undergo programmed cell death (apoptosis) prior to their desquamation from the surface [43]. However, under normal conditions, the desquamation of corneal surface cells does not require apoptosis as was once considered [9]. The living surface cells must actively dissociate themselves from neighboring cells, and we suggest that degradation of their junctional complexes is required for this to occur. We hypothesize that in the healthy cornea, surface cells are intact and alive when they desquamate from the surface and that they are able to separate from their neighboring cells because of regulated cleavage of tight junction proteins by MMPs secreted by CE cells. Conversely, in some pathological conditions, apoptosis of the surface CE layer does indeed occur [42], [44], [45].

The derangement of the CE barrier function in various corneal disorders is due to dysfunction, death, or loss of the surface layer of CE cells. For example, herpetic infection can result in stromal keratitis, leading to corneal melting, neovascularization, ulceration and perforation [1]–[3]. Over-expression of MMP occurs in CE cells after corneal damage [37], [39], [41], and MMPs are over-expressed in the CE in the areas of ulcerative keratitis that occurs in response to human immunodeficiency virus type 1 (HIV-1) infection. Over-expression of corneal MMP-2 is a characteristic of patients with ulcerative keratitis [40], [41].

The disease known as Dry Eye Syndrome (DES), or keratoconjunctivitis sicca, is a common ocular disorder in which ocular surface inflammation is considered to be both a cause and a consequence of corneal damage [1] including loss of CE barrier function. The disruption of the CE barrier function in DES is due in part to dysfunctional CE cells that lose their interconnecting tight junctions [46]. There is evidence that secretion of MMP-9 is increased in DES, and cleaves the tight junction occludin protein, resulting in breakdown of the CE barrier [47], [48]. Altered CE barrier function is the cause of ocular irritation and visual morbidity in dry eye disease, and MMP-9 plays an important role in the disruption of CE barrier function in DES.

MMP-9 knockout protects the CE from experimentally-induced dry eye desquamation and occludin breakdown in mice [48]. Compared to MMP-9 knockout mice, wild-type mice show greater desquamation of the surface CE and an increase in cleavage of occludin in the CE. Therefore, increased MMP-9 activity on the ocular surface in response to dryness disrupts CE barrier function which appears to be the result of accelerated loss of the surface CE cells mediated by proteolytic cleavage of occludin and other transmembrane junctional proteins. Therapeutic targeting of MMP-9 activity in patients with corneal erosions may be a promising route to enhance the recovery from these disorders [48]. The scenarios cited above depict a pattern of MMP participation in a range of corneal disorders in which, in response to an initial insult, MMPs convert from low constitutive expression to unchecked induced expression that causes pervasive damage to corneal tissues.

### Expression of occludin and claudin in surface corneal epithelium tight junctions

The CE surface squamous cells are joined together by tight and adhering junctions (zonula occludens and zonula adherens, respectively) on their lateral membranes. The adhering junctions provide most of the mechanical attachment between neighboring cells, and tight junction formation is preceded by and is dependent upon formation of adhering junctions [49]. The major transmembrane attachment protein of the adhering junction is cadherin, and is expressed in all layers of the CE (*i.e.*; is not specific for the surface layer) [50], which is one reason why we did not focus our attention on the potential role of MMPs in degrading adhering junctions. Disruption of adhering junctions is obviously necessary for cell dissociation, and should be a subject of future study.

Tight junctions comprise the major component of the paracellular permeability barrier as well as protection from infection and pathogen invasion in the CE. In the CE, tight junctions are present only on the surface layer of cells and are composed mostly of occludin and claudin [51]. Junctional adhesion molecule (JAM) is also a component of tight junctions, but is also expressed at cell borders throughout all layers of the CE [52], so was not a subject of this study. There are several types of claudin, and claudin-4 is one of the most consistently and highly-expressed members of the claudin family in CE [53], [54], so it was chosen for inclusion in this study. It is generally considered that occludin is ubiquitously expressed in all tight junctions, but that expression of various claudin types tends to be more tissue-specific [55]–[58]. Phosphorylation of occludin and claudin can cause changes in tight junction barrier permeability [10], [59]–[62], and may potentially contribute to dissociation of tight junction attachments. The possibility that phosphorylation of tight junction proteins contributes to the transient dissociation of surface face cells during CE desquamation may warrant further investigation.

The precedent for extracellular cleavage of transmembrane junctional proteins and subsequent degradation of barrier function by secreted MMPs has been established in many other fields of study [10]–[19], [63]. For example, there is partial co-localization of the metalloprotease meprinβ and E-cadherin at the lateral membranes in cultured MDCK cells, and Meprinβ cleaves E-cadherin extracellularly and weakens intercellular adhesion [63]. MMP-7 cleaves occludin extracellularly and increases paracellular permeability in cultured human vagina-ectocervical epithelial cells and decreases intercellular adhesion in MCDK cells [13], [64]. MMP-2 or -9 degrades claudin-5 and/or occludin in tight junctions of cultured mouse brain microvascular endothelial cells, degrading barrier integrity and increasing paracellular permeability [65]–[67]. In a hypertensive rat model, induced ischemia causes increased MMP-2 secretion and degradation claudin-5 and occludin in endothelial tight junctions, leading to breakdown of the blood-brain barrier [19].

### Expression of MMPs in the corneal epithelium

MMPs have been localized to the mammalian cornea, including the CE and tear fluid. In mice, MMP-1, 9, 10, 12, 13 and TIMP-1 RNA is up-regulated in the migrating CE after wounding, and MMP-3 protein is increased in the superficial layers of the CE after wounding [68]. In rabbits, MMP-1, 2, 7, 8 and 9 protein expression is increased in the CE after UV irradiation [69]. Most MMPs are localized diffusely throughout CE, but the MMP-7 labeling is more pronounced in the superficial layers [69].

MMP-2 and MT1-MMP (MMP-14) protein is expressed in multiple layers of the normal and keratoconus human CE, with MT1-MMP appearing mostly in the basal layer and MMP-2 more diffuse throughout the CE [70]. MMP-1, 2, 8, 9 have been shown to be present in the normal human CE [71], [72], with elevated levels of MMP-1, 2, 3, 7, 8 and 9 in melted corneas (keratolysis) [71]. RNA encoding MMP-1, 3, 9, 10, 11, and 13 are up-regulated by inflammatory cytokines in human CE [47], [72]–[75], and MMP-9 is elevated in the tear fluid of patients with dysfunctional tear syndrome [76]. MMP-9 and TIMP-1 are produced by the human CE and are present in tear fluid [72], [76]. In human tear fluid, MMP-9 is increased 200-fold and TIMP-1 is increased 4-fold upon wakening, resulting in a diurnal variation of MMP-9 activity in human tears [77]. These observations support the concept that recurrent conditions such as corneal erosions and corneal epithelial ulcerations which are exacerbated upon waking may be a result in part to increased degradative actions of MMPs that occur during the preceding night. This is also in agreement with our hypothesis that MMPs are up-regulated in a circadian fashion to cleave junctional proteins late at night to enable the surface cells to desquamate in the early morning in conjunction with the mild abrasion due to eyelid blinking.

In this report, we have shown that MMP-2, TIMP-2, and MT1-MMP proteins are expressed predominantly by the surface layer of the *Xenopus laevis* CE, and display diurnal differences in their abundance and location. Importantly, we have observed an association of MMP-2 expression with degradation of occludin and claudin-4 on the surface cell lateral membranes. The use of confocal scanning microscopy in this study provides a higher level of resolution than was afforded by the standard light or fluorescence microscopy used in previous studies. The trio of MMP-2, TIMP-2, and MT1-MMP was chosen as the focus of this study since their ternary interactions and mechanisms of action are particularly well-understood compared to most other members of the MMP family [26]–[29]. In addition to MMP-2, TIMP-2, and MT1-MMP, we also observed MMP-9 immunolabeling of the *Xenopus* CE (data not shown). There may certainly be other members of the MMP family involved in CE cell turnover, and future unbiased approaches will be likely needed for a more complete understanding of the role of MMPs in CE surface cell desquamation.

### Diurnal changes in tight junction proteins and MMP distribution in *Xenopus* corneal epithelium

In this and other studies, MMP-2 immunoreactivity is most often observed in intracytoplasmic compartments because most of the secreted enzyme is not attached to the cell at the time of fixation. Therefore, much of the MMP-2 immunoreactivity that we have observed in CE cells is more a reflection of intracellular MMP-2 synthesis and/or accumulation, than secretion and activation of the enzyme itself. However, we routinely did observe some MMP-2 localization to the CE surface cell lateral membranes, especially during the nighttime, and often co-localized with occludin or claudin-4 (Figures 4 and 5). Furthermore, this localization of MMP-2 on the lateral membranes was very often correlated to; 1) diminished tight junction protein expression, 2) separation of neighboring cell membranes, and 3) surface cell desquamation (Figures 4 and 5). Thus, MMP-2 immunoreactivity on CE lateral membranes may reflect the transient formation of the ternary complex with TIMP-2 and MT1-MMP on the plasma membrane, which would result in activation of secreted MMP-2. Our observation that MMP-2 is localized to lateral membranes far more frequently during the nighttime than during the day suggests that there is a diurnal rhythm of MMP-2 secretion and activation with highest levels occurring at nighttime in the *Xenopus* CE.

Although most of the surface CE cells exhibited intact occludin labeling at nighttime, there were many clusters of surface CE cells that lacked occludin immunoreactivity in their lateral membranes (Figures 4 and 5). We speculate that these occludin-negative surface cells are former sub-superficial layer of CE cells that had been only recently exposed to the corneal surface and had not yet begun to express occludin in their lateral membranes. We presume that the surface CE cells that were overlying these cells were very recently desquamated. In surface CE clusters bordering occludin-negative cell clusters, the occludin labeling on the lateral membranes was often disrupted. Although the cells had occludin on their lateral membranes, the cells were clearly not attached to their neighboring cells (Figure 4). This pattern suggested that the extracellular attachments between occludin molecules of neighboring cells were severed but that the occludin had not yet been internalized. MMP-2 immunoreactivity was quite elevated in both the cytoplasm and lateral membranes of these occludin-positive cells that were detached from their neighboring cells, suggestive of a causative association.

Higher levels of TIMP-2 expression in the surface CE during the day (Figure 6) is consistent with the concept of diminished MMP-2 during the day, since high levels of TIMP-2 inhibits activation of MMP-2 [26]–[29]. We speculate that after MMP-2 is secreted, and the surface cells in the area have desquamated, any residual MMP-2 needs to be inactivated to protect the newly-forming tight junctions from cleavage. TIMPs may be secreted by the former sub-superficial cells to inhibit residual MMP activity and thus provide a permissive environment for transmembrane junctional proteins in cytoplasmic compartments to move to the cell surface. The necessary orderly sequence of MMP-2 secretion and surface cell desquamation, followed by TIMP-2 secretion logically requires some temporal signals.

In contrast to the MMP-2 (and TIMP-2; see figure 6) located in intracytoplasmic compartments representing inactive pro-enzyme, intracytoplasmic MT1-MMP may represent recently activated and internalized enzyme. After maturation in the Golgi apparatus, newly-synthesized MT1-MMP is rapidly transported to the plasma membrane [78]. However, cell surface localization of MT1-MMP is often not observed immunocytochemically because the enzyme on the plasma membrane is rapidly activated and internalized into endosomal compartments [26], [78]–[80]. This is consistent with our observations that most MT1-MMP immunoreactivity is present in the cytoplasm in both the day and night (Figure 7). Conversely, inactive MT1-MMP is known to accumulate on plasma membranes presumably in anticipation of an increase in pro-MMP-2 secretion [27]. In addition to the intracytoplasmic localization of MT1-MMP, we did also observe significant MT1-MMP immunoreactivity on the lateral membranes at nighttime, but not during the day in surface CE cell clusters (Figure 7). This may represent increased accumulation of inactive MT1-MMP on the plasma membrane at nighttime to be available to activate increased pro-MMP-2 secretion at night.

Quantitative measurements of immunolabeling intensity of confocal images support the direct observations of higher MMP-2 expression at night, and lower expression levels of TIMP-2 during the day (Figure 8). Absolute expression levels of MT1-MMP were relatively unchanged during the light/dark cycle (Figure 8). As predicted, the lower levels of occludin and claudin-4 immunolabeling intensities observed in the confocal images were not strongly supported by quantitative analysis (Figure 8). As described earlier, the loss of tight junction proteins at nighttime appeared in a mosaic patchwork pattern, with most of the corneal epithelium relatively unperturbed (Figure 3). Therefore, within study groups there was wide variation in the amount of intact occludin or claudin-4, reflective of the non-uniform pattern of MMP-2 expression on the surface CE at nighttime.

### Implications for circadian regulation of corneal epithelial cell homeostasis

We suggest that tight and adhering junctions of the surface layers of the CE are assembled and dissembled on a daily basis in discrete clusters to enable surface cells to be periodically shed yet maintain the critical barrier function of the CE. The mosaic CE cell clusters display varying degrees of tight junction protein and MMP expression, but there is a strong tendency for a majority surface CE cells to exist in particular states of function at particular times of day, such as the tendency to desquamate preferentially in the early morning, compared to other the times during the diurnal cycle. Our study was intended to provide support for the hypothesis that degradation of junctional complexes, which leads to CE surface cell desquamation, is mediated via MMPs produced by CE cells which are potentially influenced by nocturnal circadian signals as part of a renewal cycle.

We have previously reported that receptors for the circadian signaling molecule melatonin are located on the lateral membranes of the *Xenopus surface* CE [81]. These observations led us to the current study and hypothesis. Furthermore, MMP-2 and the Mel1a melatonin receptor are co-localized to the surface cells of *Xenopus* CE (unpublished results). Changes in MMP-2 expression and/or activity may perhaps be a downstream consequence of nighttime melatonin receptor activation in the surface CE. Interestingly, melatonin regulates the expression and secretion of MMP-9 in several experimental systems [82]–[85], suggesting a potential mechanism for a diurnal rhythm of MMP expression and tight junction breakdown. Inhibition of MMP activity by melatonin often appears to be due to up-regulation of tissue inhibitors of MMPs (*i.e.*; TIMP-1) [85], [86], which are known to protect CE barrier function in response to infection and trauma [38], [87]–[89].

In order to maintain CE tissue homeostasis, the sequence of barrier breakdown, subsequent loss of surface cells, and re-establishment of a new surface epithelial barrier, needs to be temporally orchestrated. We propose that this sequence of events occurs preferentially during the night, when the CE has some protection by the closed eyelid during sleep. The underlying sub-superficial cells anticipate the need for forming new tight junctions by pre-accumulating tight junction proteins prior to reaching the air/surface interface [90], [91], and may be mediated by a nighttime circadian signal. Circadian regulation of MMP activity and subsequent tight junction protein cleavage may represent a novel mechanism of corneal epithelial cell turnover and renewal.
